# Knowledge, attitude, and practice of body shape and fitness among university students in China

**DOI:** 10.1186/s12889-023-16122-8

**Published:** 2023-06-22

**Authors:** Tingting Sun, Hanyue Zhang, Zhenxing Kong, Jing Yang, Xiao Jia

**Affiliations:** 1 Exercise and Physical Fitness Key Laboratory of Ministry of Education, Beijing Sport University, Beijing, 100084 China; 2 School of Physical Education, North east Normal University, Changchun, Jilin 130024 China; 3 Shuren Academy, The Affiliated High School of Peking University, Beijing, 100190 China

**Keywords:** Knowledge, Attitude, Practice, University students; body shape and fitness; China

## Abstract

**Background:**

Body shape and fitness (BSF) is critical for overall well-being, while university students in China are subjected to stress, peer pressure, performance anxiety, busy schedules, and lack of sleep, which can easily lead to poor BSF. This study aimed to explore the knowledge, attitude, and practice of BSF and related factors among university students in China.

**Methods:**

This web-based cross-sectional study was conducted on students of 15 universities in China between September 1st and November 30th, 2022. The KAP scores were evaluated using a 38-item questionnaire, including social demography, knowledge, attitude, and practice. Univariable and multivariable regression analyses were performed to identify the factors associated with KAP.

**Results:**

A total of 995 valid questionnaires were collected. There were 431 (43.3%) males and 564 (56.7%) females. Most participants were sophomores (51.2%) and freshmen (36.3%). Most participants had a BMI of 18–24 kg/m^2^ (66.1%). The students scored highly on BSF-related knowledge (8.30 ± 1.49), moderately on attitude (37.20 ± 4.46), and low on practice (19.64 ± 4.62). Multivariate logistic regression analysis showed that attitude score (*P* = 0.001), sex (*P* = 0.001), grade (*P* = 0.011), body mass index (BMI) (*P* < 0.050), parent’s education level (*P* = 0.005), monthly allowance (*P* < 0.050), and sleep quality and habits (*P* = 0.016) were independently associated with practice scores.

**Conclusions:**

University students in China were found to have good knowledge, moderate attitude, and poor practice toward BSF. Attitude, sex, grade, BMI, parents’ education, monthly living expenses, and sleep quality and habits affected their practice. More BSF-related courses or activities are required to motivate students, especially females.

**Supplementary Information:**

The online version contains supplementary material available at 10.1186/s12889-023-16122-8.

## Background

Body shape and fitness (BSF) is critical for overall well-being and can be affected by various lifestyle factors and habits (diet, level of physical activity, smoking, drinking), human biology (a person’s genetics and physiology), environment (surroundings and exposure to various factors such as sunlight or toxic substances), and healthcare services [1–3]. Body shape, posture, and appearance are valid fitness indicators [4, 5]. In addition, stress, sleep, eating behaviors, exercise, smoking, being overweight, and drinking have been well-established as factors directly impacting BSF [6–8]. Chronic diseases tend to decrease BSF; however, it remains unclear whether they should be considered when evaluating body shape and general fitness [3].

Self-management, participation, and subjectivity affect BSF and, thus, general well-being. Self-management is at the core of the BSF concept, considering that BSF is the result of an individual’s behaviors and the self-control required to implement those behaviors [9]. Participation involves deliberate and active involvement in certain activities [9], while subjectivity is reflected in the individual interpretation of the desired BSF, which can substantially vary among individuals and cultures [10]. Nevertheless, proper knowledge and behavior regarding BSF is essential to be able to manage it adequately. Physical activity benefits both physical and mental health [11–13].

Knowledge, attitude, and practice (KAP) is a structured survey method that assesses specific populations in relation to their beliefs and behaviors about a specific illness, treatment, or health-related subjects [14]. KAP studies are inexpensive, simple, and straightforward. They can be used to easily access the population of interest and obtain their view on the questions of interest [14].

University students are subjected to stress, peer pressure, performance anxiety, busy schedules, and lack of sleep [15, 16]. These can easily lead to poor life hygiene, including diets of poor nutritional value, substance abuse, and lack of exercise [17–19]. Some previously reported a poor KAP of BSF among university students, especially in relation to physical activity [20–22] and healthy foods [23]; however, no study examined the KAP in relation to BSF among university students in China. Clarifying the understanding of BSF in specific populations might help policymakers establish and implement effective fitness strategies, improving health status and quality of life and reducing morbidity and mortality [24]. In addition, a clear understanding of the association between BSF and health may promote active participation and increase the empowerment of individuals [24].

This study hypothesized that students would have proper knowledge but poor practice toward BSF. This study examined the KAP in relation to BSF among university students in China. The reported results could be used to help design and implement educational policies on BSF targeting this population.

## Methods

### Study design and participants

This cross-sectional study was conducted on students of 15 universities in Beijing (China) between September 1st and November 30th, 2022. Participants with incomplete data were excluded for analysis. It was approved by the ethics committee of Beijing Sport University (as the lead institution) and by each participating institution (2022092H). All participants provided written informed consent. The study was conducted according to the principles of the Declaration of Helsinki and the Good Clinical Practices.

We selected representative schools with regional characteristics from different regions, such as East, central, and South China. The five research assistants who received systematic training contacted the university’s student administration department and scientific research administration department and then contacted the class tutors and counselors through the student administration department. After being fully informed of the research purpose, the students were invited to complete an online questionnaire survey. Among all the contacted schools, 18 schools responded at the beginning, but later, due to the actual situation of the school’s work arrangement, it was inconvenient to survey three of them. Finally, according to the five regions in China, the universities included in this study included three schools in East China, North China, Central China, South China, and Southwest China, with 1037 students. We selected students in each grade, major, or class from 15 universities by convenience sampling to participate in the survey.

### Procedures

A four-dimensional questionnaire was designed (Additional file 1) based on the public questionnaires about BSF on the website of *WJX.cn* (an online tool to design and administer surveys, www.wjx.cn). The final questionnaire was somewhat modified according to the opinions of 8 experts and was preceded by a pre-survey with 30 questionnaires. The reliability and validity test for the questionnaire showed that Cronbach’s α was 0.858, and the Kaiser–Meyer–Olkin (KMO) index was 0.884. The final questionnaire included four dimensions and had a total of 38 items. Supplementary Table S1-2 presents the factor analysis for each question [25]. Ten items were related to the social demography information (gender, grade, body mass index (BMI), region, profession, parents’ education level, monthly living expenses, time spent in sedentary activities during the day, hunchback status, sleep status, factors affecting adherence to physical training, and methods of weight loss and body shaping. The “knowledge” dimension included 9 items, scored with 1 point for correct answers and 0 points for incorrect or unclear answers. The total scores ranged from 0 to 9 points. The “attitude” dimension included 11 items. Among them, 9 items were evaluated on a five-point Likert scale, with 5 points standing for very positive to 1 point standing for very negative. For item A7, option A was scored with 2 points, option B was scored with 1 point, option C was scored with 0 points, and the total scores ranged from 9—47 points. Item A9 was a categorical variable with five levels that were not scored with points. The “practice” dimension included 8 items, of which 5 were evaluated on a five-point Likert scale, ranging from very positive (5 points) to very negative (1 point). For item P4, option “a” was scored with 4 points, option “b” was scored with 3 points, option “c” was scored with 2 points, and option “d” was scored with 1 point. For item 5, option “a” was scored with 3 points, option “b” was scored with 2 points, and option “c” was scored with 1 point, and the total scores ranged from 7 to 32 points. Item P6 was a categorical variable with four levels that were not scored with points. High scores were determined as ≥ 80%, moderate scores as 70%-79%, and low scores as < 70%.

A questionnaire was considered valid if it was returned with one answer for each question, the answers did not follow a specific pattern (e.g., all first options), and there were no questions with multiple answers. The WeChat-based mini program was used to build online questionnaires and generate QR codes. Then, participants logged in and filled out the questionnaire by scanning the QR code. In order to ensure the quality and completeness of the questionnaire results, only one submission per IP address was allowed, and all items needed to be filled in. Members of the research team checked all questionnaires for completeness, internal consistency, and reasonableness. All questions were mandatory.

### Statistical analysis

SPSS 26.0 (IBM Corp., Armonk, N.Y., USA) was used for statistical analysis. Continuous data were expressed as means ± standard deviation and analyzed using analysis of variance (ANOVA) (The effect size were showed in Supplementary Fig. 1). Categorical data were expressed as n (%). Pearson correlation was performed to analyze the correlations between knowledge, attitude, and practice scores (The effect size were showed in Supplementary Fig. 2). Multivariable LR forward logistic regression was used to analyze the factors influencing practice scores. The median (20) of practice scores was used as the cut-off value. Variables statistically significant in univariate analysis (*P* < 0.05) were included in the multivariate logistic regression analysis. All statistical tests were two-sided, and *P*-values < 0.05 were considered statistically significant.

## Results

**Fig. 1:**
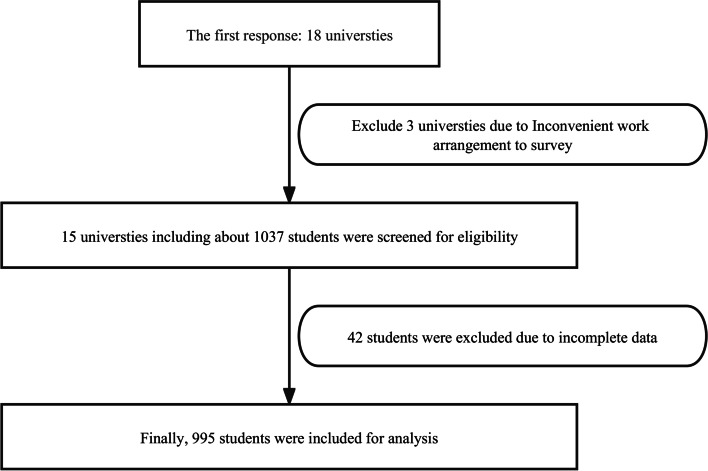
Flow chat

**Fig. 2:**
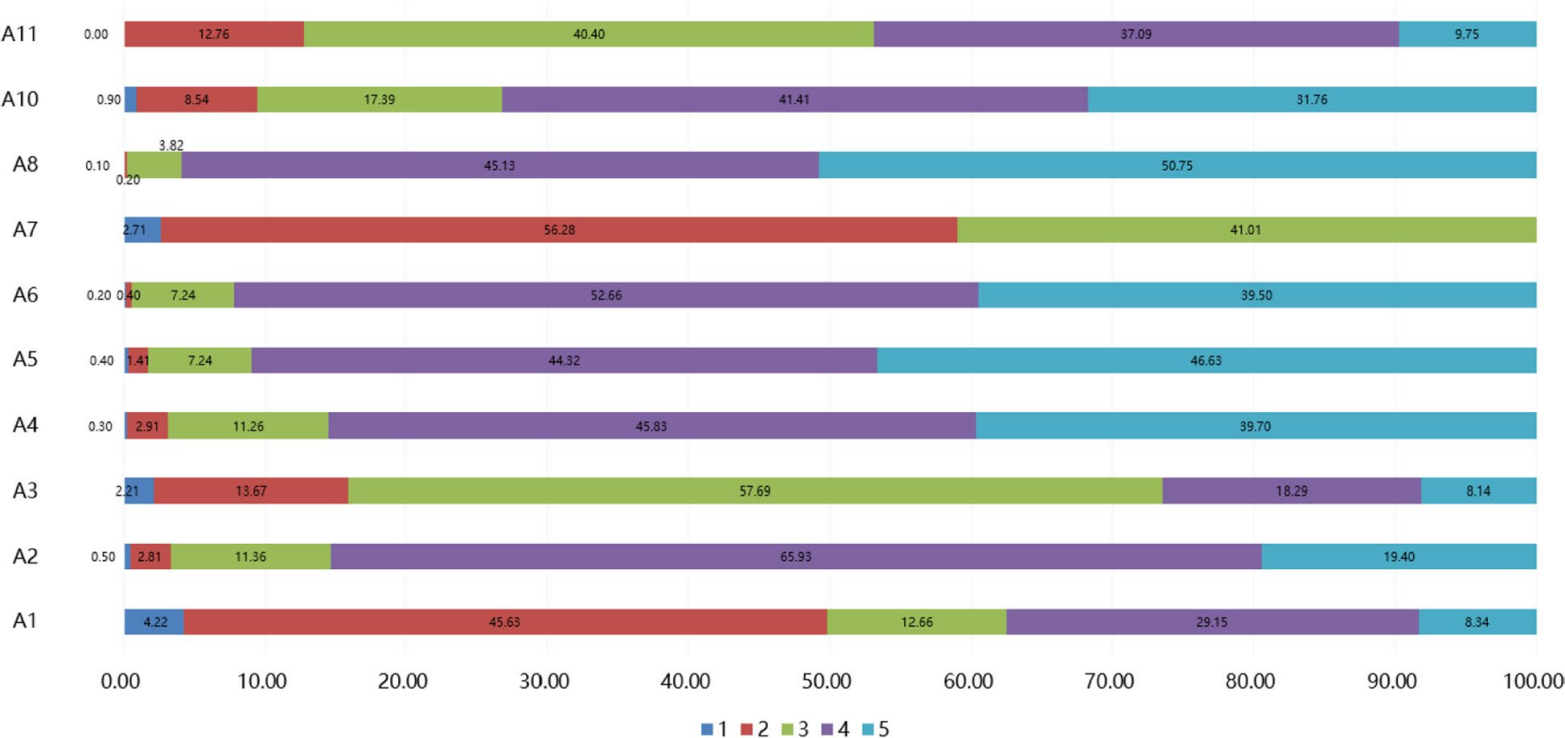
The score distribution of the “attitude” dimension. A1 ~ A11 were questions of “attitude” (Additional file 1), 1 ~ 5 were students’ score for each question

**Fig. 3:**
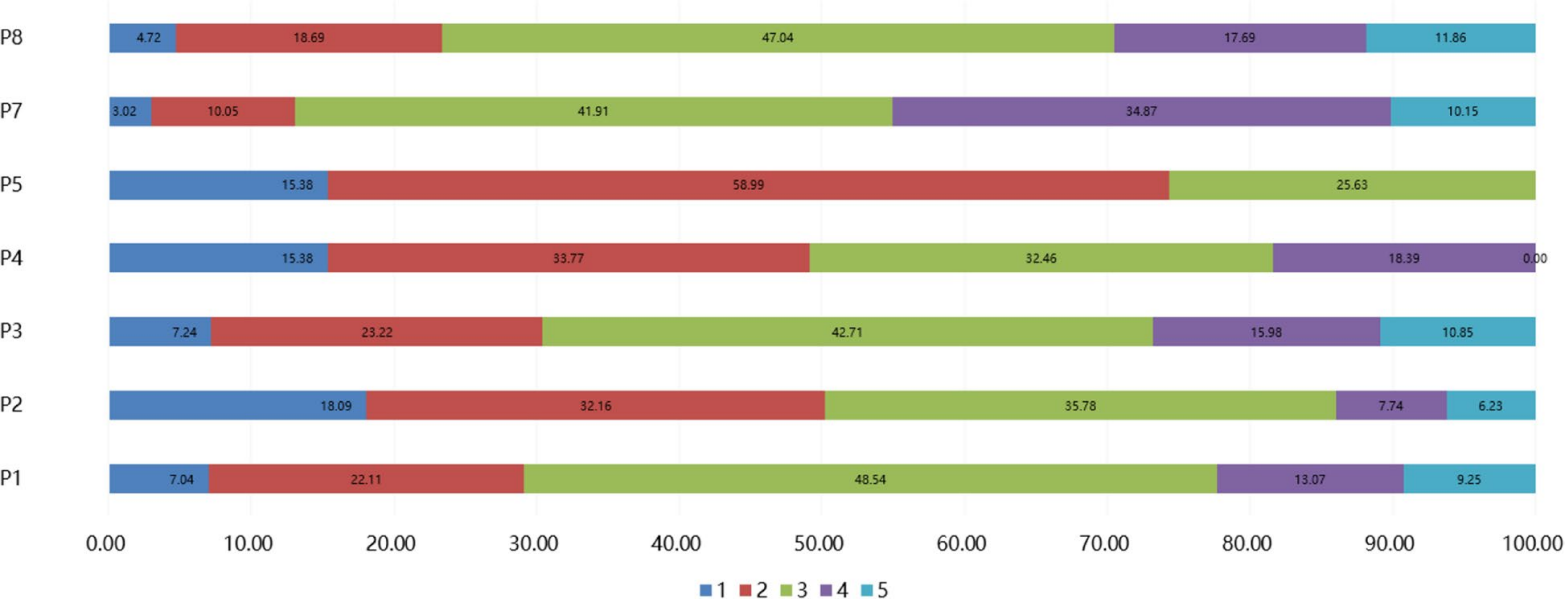
The score distribution of the “practice” dimension. A1 ~ A11 were questions of “practice” (Additional file 1), 1 ~ 5 were students’ score for each question

A total of 1035 students from 15 universities participated, 42 of who were excluded due to incomplete data. Finally, 995 valid questionnaires were included for analysis (Fig. 1). There were 431 (43.3%) male and 564 (56.7%) female participants. Most participants were sophomores (51.2%) and freshmen (36.3%). Most participants had a BMI of 18–24 kg/m^2^ and were from the Eastern (57.0%) and Central (40.1%) regions. University students scored highly on BSF-related knowledge (8.30 ± 1.49), moderately on attitude (37.20 ± 4.46), and low on practice (19.64 ± 4.62) (Table 1), which were analyzed based on the distribution of students’ knowledge (Table 2), attitude (Fig. 2), and practice (Fig. 3).

**Table 1:** Baseline characteristics and KAP scores

Variables	n (%)	Knowledge score		Attitude score		Practice score	
		Mean ± SD	*P*	Mean ± SD	P	Mean ± SD	*P*
**Total scores**	995 (100)	8.30 ± 1.49		37.20 ± 4.46		19.64 ± 4.62	
**Sex**			**0.001**		** < 0.001**		** < 0.001**
Male	431 (43.3)	8.12 ± 1.75		37.85 ± 4.73		20.59 ± 4.99	
Female	564 (56.7)	8.44 ± 1.23		36.70 ± 4.18		18.91 ± 4.17	
**Grades**			0.533		** < 0.001**		**0.002**
Freshman	361 (36.3)	8.37 ± 1.28		37.73 ± 4.03		19.89 ± 4.42	
Sophomore	509 (51.2)	8.25 ± 1.58		36.51 ± 4.67		19.21 ± 4.76	
Junior	87 (8.7)	8.37 ± 1.38		38.20 ± 4.34		20.11 ± 4.33	
Senior	9 (0.9)	7.11 ± 3.14		38.33 ± 4.42		23.00 ± 5.39	
Undergraduate and graduate students	29 (2.9)	8.48 ± 1.62		39.45 ± 4.26		21.59 ± 4.01	
**BMI**			0.075		**< 0.001**		**0.012**
< 18	167 (16.8)	8.13 ± 1.86		37.32 ± 4.83		18.75 ± 4.73	
18–24	658 (66.1)	8.39 ± 1.26		37.50 ± 4.30		19.92 ± 4.57	
> 24	170 (17.1)	8.14 ± 1.82		35.94 ± 4.51		19.42 ± 4.60	
**Region**			0.481		0.416		0.405
Northern region	2 (0.2)	9.00 ± 0.00		41.50 ± 3.54		25.00 ± 4.24	
Eastern region	567 (57.0)	8.24 ± 1.62		37.31 ± 4.36		19.63 ± 4.52	
Central region	399 (40.1)	8.38 ± 1.32		37.04 ± 4.56		19.64 ± 4.72	
Western region	27 (2.7)	8.37 ± 0.93		36.93 ± 5.01		19.22 ± 5.09	
**Profession**			** < 0.001**		**0.007**		** < 0.001**
Biomedicine	1 (0.1)	0.00		28.00 ± 0.00		18.00 ± 0.00	
Art and sports	136 (13.7)	8.18 ± 1.68		38.04 ± 4.68		21.33 ± 4.96	
Other	858 (86.2)	8.33 ± 1.43		37.08 ± 4.40		19.37 ± 4.50	
**Parents’ education level**			0.457		**0.012**		** < 0.001**
High school and below	713 (71.7)	8.30 ± 1.45		36.91 ± 4.29		19.21 ± 4.43	
University/Junior University	261 (26.2)	8.34 ± 1.50		37.82 ± 4.64		20.65 ± 4.79	
Graduate or above	21 (2.1)	7.71 ± 2.26		39.14 ± 6.31		21.48 ± 6.21	
**Monthly allowance**			0.122		**0.007**		**0.001**
< 1500	279 (28.0)	8.16 ± 1.54		36.61 ± 4.32		18.81 ± 4.27	
1500–2000	556 (55.9)	8.40 ± 1.37		37.23 ± 4.54		19.76 ± 4.61	
2000–3000	140 (14.1)	8.26 ± 1.56		38.03 ± 4.24		20.62 ± 4.99	
> 3000	20 (21.1)	7.70 ± 2.755		38.70 ± 4.612		20.90 ± 4.919	
**Duration of the sitting state every day**			0.878		** < 0.001**		** < 0.001**
< 5 h	255 (25.6)	8.34 ± 1.52		38.17 ± 4.44		21.10 ± 4.80	
5–8 h	534 (53.7)	8.31 ± 1.39		36.89 ± 4.17		19.31 ± 4.24	
8–10 h	153 (15.4)	8.27 ± 1.51		36.46 ± 4.68		18.20 ± 4.53	
> 10 h	53 (5.3)	8.17 ± 2.10		37.75 ± 5.84		20.00 ± 5.71	
**Status of hunchback**			0.252		** < 0.001**		** < 0.001**
Yes	419 (42.1)	8.35 ± 1.47		36.42 ± 4.19		18.88 ± 4.30	
No	400 (40.2)	8.34 ± 1.36		38.51 ± 4.52		20.92 ± 4.76	
Unclear	176 (17.7)	8.11 ± 1.77		36.09 ± 4.23		18.54 ± 4.34	
**Status of sleep**			0.138		** < 0.001**		** < 0.001**
Good sleep quality and habits	454 (45.6)	8.38 ± 1.29		38.02 ± 4.34		20.58 ± 4.50	
Good sleep quality and poor habits	329 (33.1)	8.31 ± 1.58		36.75 ± 4.48		18.98 ± 4.59	
Insomnia	212 (21.3)	8.12 ± 1.71		36.15 ± 4.37		18.64 ± 4.52	
**Factors that affect your adherence to physical training**							
Poor self-coordination	616 (61.9)	8.36 ± 1.36	0.135	37.21 ± 4.61	0.932	19.56 ± 4.73	0.529
Difficult movement	528 (53.1)	8.27 ± 1.569	0.424	36.85 ± 4.191	**0.008**	19.41 ± 4.372	0.111
Exaggerated action	240 (24.1)	8.29 ± 1.576	0.897	37.02 ± 4.423	0.465	19.90 ± 4.628	0.311
Lack of interest	453 (45.5)	8.22 ± 1.533	0.095	36.45 ± 4.451	** < 0.001**	18.93 ± 4.517	** < 0.001**
Other	89 (8.9)	8.12 ± 1.770	0.234	37.27 ± 4.258	0.877	19.56 ± 4.767	0.872
**Methods of weight loss and body shaping**			7.643	0.025		0.150	0.101
Diet	199 (20.0)	8.48 ± 1.32		36.71 ± 5.29		19.32 ± 5.83	
Fitness/Yoga/Body Classes/Dance	728 (73.2)	8.33 ± 1.41		37.42 ± 4.15		19.83 ± 4.24	
Diet pill	9 (0.9)	7.22 ± 2.59		35.11 ± 7.41		19.11 ± 2.67	
Other	59 (5.9)	7.56 ± 2.26		36.47 ± 4.40		18.39 ± 4.52

**Table 2:** The “Knowledge” dimension

**Knowledge (Appendix)**	False or Don’t know, n (%)	Right, n (%)
K1. Figure refers to the body shape and appearance. A healthy body needs to have internal and external harmony, be well-proportioned and in the correct shape, and have a straight posture	71 (7.1)	924 (92.9)
K2. Health management emphasizes the management of daily life patterns and believes that early health management leads to better outcomes. Also, the more scientific and persistent health behaviors are implemented in daily life, the better the outcomes	74 (7.4)	921 (92.6)
K3. BSF management mainly include nutritional diet management, sports and fitness management, psychological and emotional management, and auxiliary aesthetical management	90 (9.0)	905 (91.0)
K4. Different exercise styles have different effects on body shape	27 (2.7)	968 (97.3)
K5. The basic postures refer to standing, sitting, walking, and lying	64 (6.4)	931 (93.6)
K6. Poor body posture includes round shoulders, right-angled shoulders, high and low shoulders, hunchback, head forward tilt, pelvic forward tilt, O-leg, X-leg, spinal deformity, being too obese, and being too thin	89 (8.9)	906 (91.1)
K7. Scoliosis, commonly known as scoliosis, is a three-dimensional spine deformity that includes sequence abnormalities in coronal, sagittal, and axial positions	118 (11.9)	877 (88.1)
K8. People who are not physically healthy are more likely to suffer from chronic diseases	108 (10.9)	887 (89.1)
K9. Long-term poor posture can cause physical problems, such as hunchback, spinal curvature, etc., and can also contribute to compression and damage internal organs	53 (5.3)	942 (94.7)

Furthermore, Pearson correlation analyses revealed positive correlations between the attitude and knowledge scores (*r* = 0.211, *P* < 0.001), practice and knowledge scores (*r* = 0.148, *P* < 0.001), and attitude and practice scores (*r* = 0.665, *P* < 0.001) (Table 3). Multivariate logistic regression analysis revealed that the attitude scores (OR = 1.35, 95%CI: 1.29–1.41, *P* = 0.001), female (OR = 0.57, 95%CI: 0.41–0.80, *P* = 0.001), sophomore of grade (vs. freshman, OR = 1.60, 95%CI: 1.11–2.30, *P* = 0.011), BMI of 18–24 kg/m^2^ (vs. < 18 kg/m^2^, OR = 1.71, 95%CI: 1.12–2.61, *P* = 0.013), BMI of > 24 kg/m^2^ (vs. < 18 kg/m^2^, OR = 2.17, 95%CI: 1.25–3.76, *P* = 0.006), parents with university/junior university educational levels (vs. high school and below, OR = 1.69, 95%CI: 1.18–2.42, *P* = 0.005), monthly allowance of 1500–2000 yuan (vs. < 1500 yuan, OR = 1.76, 95%CI: 1.24–2.49, *P* = 0.002), monthly allowance of 2000–3000 yuan (vs. < 1500 yuan, OR = 1.88, 95%CI: 1.14–3.11, *P* = 0.014), and poor sleep habits (vs. good sleep habits, OR = 0.66, 95%CI: 0.47–0.92, *P* = 0.016) were independently associated with higher practice scores (> 20) (Table 4).

**Table 3:** Pearson correlation analysis

	Knowledge	Attitude	Practice
Knowledge	1		
Attitude	0.211 (*P* < 0.001)	1	
Practice	0.148 (*P* < 0.001)	0.665 (*P* < 0.001)	1

**Table 4:** Multivariable logistic regression analysis for factors associated with a score of practice > 20

Factors	Univariate logistic regression		Multivariate logistic regression	
	OR (95%CI)	*P*	OR (95%CI)	*P*
**Knowledge score**	1.155 (1.055–1.265)	0.002		
**Attitude score**	1.352 (1.294–1.412)	** < 0.001**	1.353 (1.291–1.418)	**0.001**
**Sex**				
Male	REF		REF	
Female	0.503 (0.390–0.649)	** < 0.001**	0.574 (0.411–0.804)	**0.001**
**Grades**				
Freshman	REF		REF	
Sophomore	0.735 (0.5610–0.963)	**0.026**	1.598 (1.111–2.299)	**0.011**
Junior	0.995 (0.623–1.590)	0.984	1.141 (0.651–2.001)	0.645
Senior	3.404 (0.689–16.610)	0.130	5.367 (0.945–30.483)	0.058
Undergraduate and graduate students	1.848 (0.836–4.084)	0.129	2.326 (0.909–5.951)	0.078
**BMI**				
< 18	REF		REF	
18–24	1.580 (1.117–2.237)	**0.010**	1.710 (1.120–2.612)	**0.013**
> 24	1.500 (0.972–2.313)	0.067	2.171 (1.254–3.758)	**0.006**
**Parents’ education level**				**0.013**
High school and below	REF		REF	
University/Junior University	1.903 (1.427–2.538)	** < 0.001**	1.686 (1.175–2.420)	**0.005**
Graduate or above	1.455 (0.610–3.469)	0.398	0.748 (0.228–2.459)	0.633
**Monthly allowance**				
< 1500	REF		REF	
1500–2000	1.748 (1.301–2.347)	** < 0.001**	1.757 (1.238–2.494)	**0.002**
2000–3000	2.247 (1.486–3.398)	** < 0.001**	1.881 (1.139–3.108)	**0.014**
> 3000	2.603 (1.030–6.579)	**0.043**	1.730 (0.557–5.375)	0.343
**Duration of the sitting state every day**				
< 5 h	REF			
5–8 h	0.613 (0.453–0.828)	0.001		
8–10 h	0.383 (0.253–0.581)	0.000		
> 10 h	0.696 (0.385–1.260)	0.232		
**Status of hunchback**				
Yes	REF			
No	2.044 (1.548–2.701)	< 0.001		
Unclear	0.891 (0.621–1.279)	0.533		
**Status of sleep**				
Good sleep quality and habits	REF		REF	
Good sleep quality and poor habits	0.569 (0.427–0.758)	** < 0.001**	0.655 (0.465–0.924)	**0.016**
Insomnia	0.477 (0.342–0.666)	** < 0.001**	0.747 (0.497–1.122)	0.160

## Discussion

The results of the present study showed that university students in China had a high knowledge of BSF, moderate attitude, and low participation. Also, the attitude, sex, grade, BMI, parent’s educational level, monthly allowance, and sleep habits were independently associated with practice scores. The reported results might provide useful cues for universities to develop reasonable interventions to improve BSF-related attitudes and practices among university students.

In the KAP concept, knowledge, attitude, and practice are interrelated since more knowledge leads to a better attitude, which is conducive to better practice [26]. In addition, people with a better attitude and/or practice toward a certain subject are more likely to seek more knowledge [14]. In this study, all three scores were correlated, but only the attitude scores were independently associated with the practice scores. Nevertheless, these results indicated that university students had high levels of knowledge of BSF; however, their attitude and practice might require improvement. Indeed, BSF is essentially based on self-management [9], and improving the KAP toward BSF should also improve self-management. Therefore, future programs should focus on improving these two aspects.

In the present study, women had lower practice scores toward BSF. A similar result was reported on physical activity in India [22]. A survey conducted in Australian universities suggested that 90% of men and 82% of women met the criteria suggested by the physical activity guidelines [27]. A study on nursing students suggested that some types of physical activity offered in schools and universities might be more attractive to male students than to female ones [28]. Disparities between sexes in offering services related to BSF in universities should be explored. Future studies should address these issues.

In the present study, sophomores had a higher frequency of high practice scores than freshmen, which could relate to integrating and habituating to the university rhythm of life and allowing more time for BSF. This relationship was also observed by Trockel et al*.* [29] regarding physical activity.

A better BMI was also related to a higher frequency of high practice scores. Previous studies reported that high BMI is related to higher physical performance [30, 31], consistent with the present study. However, some studies also report the contrary [32, 33]. Still, this relationship must be taken with caution. Indeed, students with a higher BMI might be more willing to perform activities to acquire better BSF habits and a better physical image as they might be more aware of the health risks associated with a high BMI [34]. Also, muscle tissue is heavier than adipose tissue, and many people with good physical fitness tend to have a high BMI without having much adipose tissue [35]. Universities in China and elsewhere currently propose several physical activities and team sports which could increase BMI in students with good BSF practices.

A higher parental education level is conducive to better health literacy in their children [36], leading to better BSF KAP. Higher monthly expenses are also associated with a better socioeconomic status, predicting health KAP [37, 38]. Sleep quality and good sleep habits are related to various health outcomes, including diabetes, cardiovascular disease, depression, anxiety, and obesity [39–41]. Hence, maintaining good sleep quality constitutes good life hygiene and is conducive to better practice of good BSF habits.

### Strengths and limitations

The present study has some strengths. While previous studies often focused on a single aspect of BSF (e.g., physical activity or diet), the present study focused on BSF. Indeed, BSF is a complex concept where all constituents are interrelated and influence each other. Although isolating a single factor can provide useful information, such an approach fails to elucidate the concept fully. The approach used in the present study might be considered too broad; nevertheless, it still provides data on factors that should be paid attention to in the comprehensive context of all other parameters involved in BSF. Nevertheless, the present study has some limitations. Despite being a multicenter study, the sample size was relatively small when considering the number of university students in China. In addition, the Northern and Western regions were underrepresented. The questionnaire was self-designed according to the reality and characteristics of the students, and its generalizability to other populations is unknown. Finally, the factor analysis did not show quite good construct validity of the questionnaire for the data. On this basis, researches with more rigorous data quality control will be carried out.

## Conclusions

In conclusion, university students in China showed good knowledge, moderate attitude, and poor practice of BSF. Multiple factors, including attitude scores, sex, grade, BMI, parent’s education level, monthly allowance, and sleep habits, were independently associated with practice scores. Therefore, universities should provide more BSF-related courses or activities to motivate students, especially women, to improve their BSF-related practices.

## Supplementary Information

**Additional file 1.** The questionnaire.

**Additional file 2:** **Supplementary Table S1.** Factor analysis for each question. **Supplementary Table S2.** Fitting index of factor analysis. **SupplementaryFigure 1** Effect size for Table 1. **Supplementary Figure 2** Effect size for Pearson correlation analysis.

## Data Availability

All data generated or analyzed during this study are included in this published article.

## Abbreviations

BSF: Body shape and fitness
KAP: Knowledge, Attitude, and Practice
KMO: Kaiser-Meyer-Olkin
BMI: Body mass index
