# Supplementary material for: Knowledge, attitude, and practice of body shape and fitness among university students in China
Source: BMC Public Health. 2023 Jun 22;23:1208. doi: 10.1186/s12889-023-16122-8 (PMC10286452; doi:10.1186/s12889-023-16122-8)
Supplement: Supplementary file 1 — Additional file 1. The questionnaire. [file 12889_2023_16122_MOESM1_ESM.docx]

**Additional file 1:** The questionnaire.

| Dear student,  We are from a research group of Practical Education of Body Shape and Fitness (BSF) from Beijing Sport University, and this questionnaire is designed to investigate the knowledge, attitude, and practice of college students on BSF. The information collected in this questionnaire is confidential, and please be assured that your information will not be compromised. To guarantee the accuracy and validity of this survey, please answer the questions according to your real conditions as possible. Thank you very much for making time to participate in this study. We sincerely thank you for your support and cooperation in this study.  □ I understand and consent that the data collected in this study will be used for scientific researches. |
| --- |

| **Part 1 General Information** | | | |
| --- | --- | --- | --- |
| 1. Sex: | a. Male | | b. Female |
| 2. Age: years, Grade: . | | | |
| 3. Height: (cm), body weight: (kg). | | | |
| 4. Region where you currently live in: . | | | |
| 5. Your major: . | | | |
| 6. What are your parents’ educational levels? | a. Senior middle school or lower  b. Junior college/college  c. Postgraduate or higher | | |
| 7. How about your monthly allowance (Yuan)? | a.<1500  b.1500~2000  c.2000~3000  d.3000~5000  e.>5000 | | |
| 8. The time that you are in sitting position every day ｡ | | a. <5 h  b. 5-8 h  c. 8-10 h  d. >10 h | |
| 9. Do you have conditions like hunchback? | | a. Yes  b. No  c. Unclear | |
| 10. Please describe your sleep condition. | | a. High sleep quality and good sleep habit  b. High sleep quality but poor sleep habit  c. Insomnia | |

| **Part 2 Knowledge on BSF** | |
| --- | --- |
| K1. Body shape refers to body morphology and image, and healthy body shape requires getting refined internally and externally to acquire healthy body, well-proportioned and symmetric morphology, full and soft figure, and elegant dynamic posture.  a. Yes b. No c. Unclear |  |
| K2. Health management highlights the management of daily lifestyle; the earlier initiation of health management, and more scientific and persistent of healthy behaviors in daily livings could better benefit our health.  a. Yes b. No c. Unclear |  |
| K3. BSF management mainly include various aspects such as nutrition and diet management, exercise and fitness management, psychological and emotional management, accessory management by bodybuilding underwear.  a. Yes b. No c. Unclear |  |
| K4. The effects of different modes of exercises on body morphology vary.  a. Yes b. No c. Unclear |  |
| K5. The basic postures of human include stand, sit, walk, and lie down.  a. Yes b. No c. Unclear |  |
| K6. Poor body postures include round shoulder, right-angle shoulder, high and low shoulders, hunchback, forward head posture, anterior pelvic tilt, genu varum, genu valgum, spinal deformation, over-obese, and over-thin.  a. Yes b. No c. Unclear |  |
| K7. Scoliosis, also known as lateral spinal flexion, is a 3-dimensional deformity of spine that includes abnormalities on the coronal, sagittal, and axial sequences.  a. Yes b. No c. Unclear |  |
| K8. People with poor BSF have higher risks of chronic diseases.  a. Yes b. No c. Unclear |  |
| K9. Long-term poor body morphology could lead to BSF conditions such as hunchback and spinal curvature, as well as compression and injuries to viscera.  a. Yes b. No c. Unclear |  |

| **Part 3 Attitude on BSF** |
| --- |
| A1. Are you satisfactory to your body shape?  a. Highly satisfactory; b. Satisfactory; c. Fair; d. Unsatisfactory; e. Highly unsatisfactory |
| A2. Do you care your body shape and figure?  a. Very much; b. A bit; c. Neutral; d. Not so much; d. Not at all |
| A3. How do you rate your body posture?  a. Very nice; b. Nice; c. Fair; d. Poor; e. Very poor |
| A4. Are you willing to enhance physical exercises to become healthier?  a. Highly agree; b. Agree; c. Neutral; d. Disagree; e. Highly disagree |
| A5. People with good figures and health status are more popular in socialization.  a. Highly agree; b. Agree; c. Neutral; d. Disagree; e. Highly disagree |
| A6. Are you willing to correct the poor posture or shape habits intentionally?  a. Very much; b. Yes; c. Neutral; d. No; e. Highly unwilling |
| A7. What will you do if examinations show you have spinal shape and fitness issues?  a. Worry very much; b. A bit worry; c. Neutral |
| A8. Shape-up exercise could help improving the poor body posture.  a. Highly agree; b. Agree; c. Neutral; d. Disagree; e. Highly disagree |
| A9. Which factors do you think could influence you in sticking to shape-up exercises?  a. Poor coordination of yourself; b. High difficulty of the movements; c. Exaggerating movements; d. Lack of interests to what you learned; e. Others |
| A10. Are you willing to participate in in/out school activities of fitness training/fat reduction?  a. Very much; b. Yes; c. Neutral; d. No; e. Highly unwilling |
| A11. What’s your opinion on the healthy degree of the lifestyles of modern students?  a. All of them are healthy; b. Most of them are relatively healthy; c. A small part of them are relatively healthy; d. They are generally unhealthy |

| **Part 4 Practice on BSF** |
| --- |
| P1. Do you do BSF management in spare time?  a. Always; b. Often; c. Sometimes; d. Rarely; e. Never |
| P2. I will spend some money on BSF management.  a. Always; b. Often; c. Sometimes; d. Rarely; e. Never |
| P3. I will pay attention to and try new BSF management methods.  a. Always; b. Often; c. Sometimes; d. Rarely; e. Never |
| P4. What’s your frequency of exercises?  a. Every day; b. ≥3 times per week; c. <3 times per week; d. Barely |
| P5. What’s the status of breakfast, vegetables, fruits, coarse food grain, dairy products, and meat for you?  a. Highly taken care of nutritional balance in three meals every day; b. Sometimes take care of it; c. Never take care of it. |
| P6. What is your preferred measurement of losing weight and shaping up?  a. On diet; b. Fitness/yoga/shaping lessons/dance; c. Weight-reducing aid; d. Others |
| P7. Will you change your body shape due to others’ opinions?  a. High consistent; b. Consistent; c. Fair; d. Inconsistent; e. Highly inconsistent |
| P8. Will you pay attention to knowledge of spinal morphology?  a. Always; b. Often; c. Sometimes; d. Rarely; e. Never |
