# Supplementary material for: Knowledge, attitude, and practice of body shape and fitness among university students in China
Source: BMC Public Health. 2023 Jun 22;23:1208. doi: 10.1186/s12889-023-16122-8 (PMC10286452; doi:10.1186/s12889-023-16122-8)
Supplement: Supplementary file 2 — Additional file 2: Supplementary Table S1. Factor analysis for each question. Supplementary Table S2. Fitting index of factor analysis. SupplementaryFigure 1 Effect size for Table 1. Supplementary Figure 2 Effect size for Pearson correlation analysis. [file 12889_2023_16122_MOESM2_ESM.docx]

**Supplementary Tables and Figures**

**Supplementary Table S1.** Factor analysis for each question.

| Dimension | Item | Coefficient | Standard error | *z* | *p* | Standard estimate |
| --- | --- | --- | --- | --- | --- | --- |
| K | K1 | 1.000 | - | - | - | 0.409 |
| K | K2 | 1.174 | 0.121 | 9.701 | 0.000 | 0.471 |
| K | K3 | 1.617 | 0.150 | 10.786 | 0.000 | 0.594 |
| K | K4 | 0.762 | 0.077 | 9.938 | 0.000 | 0.494 |
| K | K5 | 1.216 | 0.119 | 10.202 | 0.000 | 0.522 |
| K | K6 | 1.598 | 0.149 | 10.757 | 0.000 | 0.590 |
| K | K7 | 2.081 | 0.184 | 11.318 | 0.000 | 0.678 |
| K | K8 | 1.896 | 0.171 | 11.110 | 0.000 | 0.642 |
| K | K9 | 1.316 | 0.120 | 10.952 | 0.000 | 0.618 |
| A | A1 | 1.000 | - | - | - | 0.222 |
| A | A9b | -0.183 | 0.074 | -2.480 | 0.013 | -0.091 |
| A | A9c | -0.084 | 0.060 | -1.385 | 0.166 | -0.048 |
| A | A9d | -0.349 | 0.086 | -4.070 | 0.000 | -0.173 |
| A | A9e | 0.065 | 0.041 | 1.611 | 0.107 | 0.057 |
| A | A10 | 2.458 | 0.384 | 6.404 | 0.000 | 0.637 |
| A | A11 | 0.853 | 0.170 | 5.015 | 0.000 | 0.253 |
| A | A2 | 1.496 | 0.239 | 6.267 | 0.000 | 0.543 |
| A | A3 | 1.313 | 0.225 | 5.842 | 0.000 | 0.387 |
| A | A4 | 2.313 | 0.356 | 6.499 | 0.000 | 0.735 |
| A | A5 | 1.640 | 0.260 | 6.314 | 0.000 | 0.570 |
| A | A6 | 1.985 | 0.304 | 6.524 | 0.000 | 0.771 |
| A | A7 | 1.031 | 0.169 | 6.119 | 0.000 | 0.473 |
| A | A8 | 1.448 | 0.228 | 6.366 | 0.000 | 0.606 |
| A | A9a | -0.040 | 0.067 | -0.588 | 0.557 | -0.020 |
| P | P1 | 1.000 | - | - | - | 0.815 |
| P | P2 | 1.062 | 0.038 | 27.840 | 0.000 | 0.811 |
| P | P3 | 1.073 | 0.038 | 28.530 | 0.000 | 0.828 |
| P | P4 | 0.588 | 0.038 | 15.640 | 0.000 | 0.499 |
| P | P5 | 0.371 | 0.025 | 14.947 | 0.000 | 0.479 |
| P | P6 | -0.001 | 0.027 | -0.019 | 0.985 | -0.001 |
| P | P7 | 0.399 | 0.036 | 10.946 | 0.000 | 0.358 |
| P | P8 | 0.702 | 0.039 | 18.168 | 0.000 | 0.569 |

**Supplementary Table S2.** Fitting index of factor analysis.

| Fitting index | | | | | | | | | | |
| --- | --- | --- | --- | --- | --- | --- | --- | --- | --- | --- |
| Common index | χ2 | *df* | *p* | χ2/*df* | GFI | RMSEA | RMR | CFI | NFI | NNFI |
| Criterion of judgment | - | - | >0.05 | <3 | >0.9 | <0.10 | <0.05 | >0.9 | >0.9 | >0.9 |
| Value | 2584.905 | 461 | 0.000 | 5.607 | 0.846 | 0.068 | 0.044 | 0.757 | 0.720 | 0.739 |
| Other index | TLI | AGFI | IFI | PGFI | PNFI | SRMR | RMSEA 90% CI |  |  |  |
| Criterion of judgment | >0.9 | >0.9 | >0.9 | >0.9 | >0.9 | <0.1 | - |  |  |  |
| Value | 0.739 | 0.824 | 0.758 | 0.739 | 0.669 | 0.069 | 0.061 ~ 0.071 |  |  |  |
| Default Model: χ2(496)=9241.622, *p*=1.000 | | | | | | | | | | |


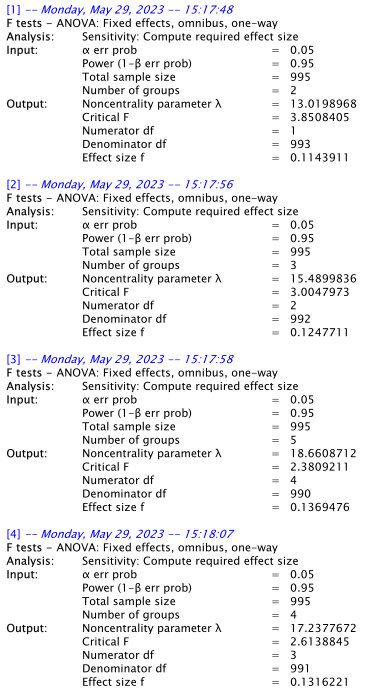


**Supplementary** **Figure 1** Effect size for Table 1.


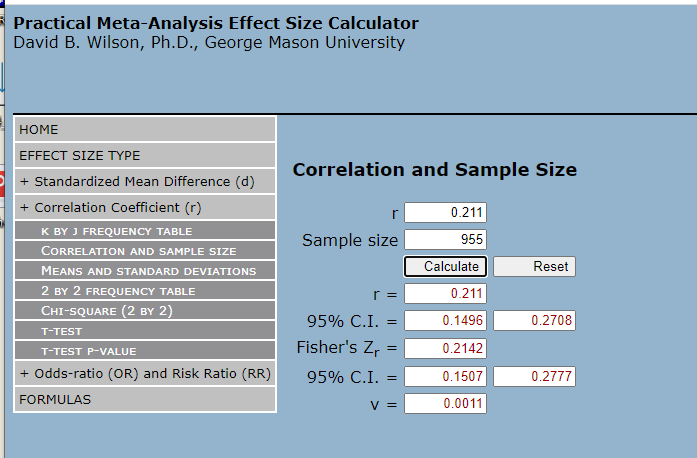


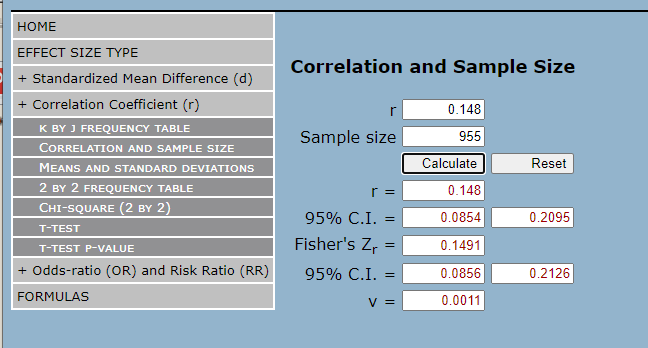


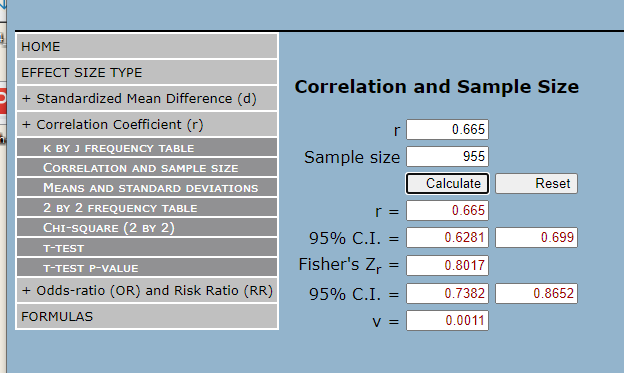


**Supplementary** Figure 2 Effect size for Pearson correlation analysis.
